# Instrumental activities of daily living in older patients with metastatic prostate cancer: results from the meet-URO network ADHERE prospective study

**DOI:** 10.1038/s41598-024-53581-4

**Published:** 2024-02-28

**Authors:** Lucia Fratino, Jerry Polesel, Emilio Francesco Giunta, Marco Maruzzo, Sebastiano Buti, Mona Ali Hassan, Umberto Basso, Sara Elena Rebuzzi, Ugo De Giorgi, Marika Cinausero, Helga Lipari, Teresa Gamba, Davide Bimbatti, Arianna Dri, Paola Ermacora, Francesca Vignani, Giuseppe Fornarini, Pasquale Rescigno, Giuseppe Luigi Banna

**Affiliations:** 1 Department of Medical Oncology, Centro di Riferimento Oncologico di Aviano (CRO) IRCCS, Aviano, Italy; 2 Unit of Cancer Epidemiology, Centro di Riferimento Oncologico di Aviano (CRO) IRCCS, Aviano, Italy; 3 Department of Medical Oncology, IRCCS Istituto Romagnolo per lo Studio dei Tumori (IRST) “Dino Amadori”, Meldola, Italy; 4 Medical Oncology 1 Unit, Department of Oncology, Istituto Oncologico Veneto IOV IRCCS, Padua, Italy; 5 Oncology Unit, University Hospital of Parma, Parma, Italy; 6 Department of Medicine and Surgery, University of Parma, Parma, Italy; 7 Portsmouth Hospitals University NHS Trust, Portsmouth, PO6 3LY UK; 8 Faculty of Science and Health, School of Pharmacy and Biomedical Sciences, University of Portsmouth, Portsmouth, PO1 2UP UK; 9 Medical Oncology Unit, Ospedale San Paolo, Savona, Italy; 10 Department of Internal Medicine and Medical Specialties (Di.M.I.), University of Genova, Genoa, Italy; 11 Department of Oncology, ASUFC Santa Maria Della Misericordia, Udine, Italy; 12 Division of Medical Oncology, Cannizzaro Hospital, Catania, Italy; 13 Medical Oncology, Mauriziano Hospital, Turin, Italy; 14 Department of Medicine, University of Udine, Udine, Italy; 15 Medical Oncology Unit 1, IRCCS Ospedale Policlinico San Martino, Genoa, Italy; 16 Candiolo Cancer Institute, FPO-IRCCS, SP142, km 3,95, 10060 Candiolo, Turin Italy

**Keywords:** Urological cancer, Cancer

## Abstract

Instrumental activities of daily living (IADL) are significant health indicators closely related to executive functions and able to detect mild cognitive impairment. A decline in IADL usually precedes ADL limitation, including taking medications, and may therefore predict a cognitive decline. We aimed to investigate the association of patients’ IADL score with other clinical factors, with a particular focus on the presence of a caregiver, and the impact on adherence to androgen receptor pathway inhibitors (ARPIs) and survival outcomes within the Meet-URO 5—ADHERE study. It was a large prospective multicentre observational cohort study monitoring adherence to ARPIs in 234 metastatic castrate-resistant PC (mCRPC) patients aged ≥ 70. We observed an association between impaired IADL and lower geriatric G8 scores (p < 0.01), and lower adherence to ARPIs whether assessed by pill counting (p = 0.01) or self-reported by the patient himself (p = 0.03). The combination of an IADL < 6 and the absence of a caregiver resulted in a significantly high risk of non-adherence to the ARPIs at the multivariable analysis (HR 9.23, 95% confidence interval 2.28–37.43, p = 0.01). IADL alongside the geriatric G8 scales represent essential tools to identify frail and less auto-sufficient patients who are extremely vulnerable particularly if not supported by a caregiver and have the highest risk of nonadherence to ARPIs.

## Introduction

Prostate cancer is the most frequent cancer among men older than 50 years, accounting for nearly a quarter of all new cancer diagnoses in men in Western countries^1^*.* The mean age at diagnosis of prostate cancer is 68 years^2^. However, the metastatic disease requiring cancer treatment might occur up to 10–15 years from diagnosis, therefore, hitting a much older population^3^.

In recent years, the use of novel androgen receptor pathway inhibitors (ARPIs) for the treatment of metastatic prostate cancer has been particularly successful in older patients due to their easy administration, manageability and favourable toxicity profile. ARPIs, however, are often associated with various forms of cognitive impairments, a condition that is already common in the older and frail population due to the elevated frequency of neurological, metabolic or vascular comorbidities^4^. Furthermore, regarding the use of oral agents like ARPIs, adherence plays an important role in patients who must self-manage and self-administer the medication. In fact, adherence to ARPIs may be associated with favourable treatment outcomes, whilst inadequate intake is potentially linked to a lack of efficacy and drug resistance^5,6^.

In a previous investigation of our study group, we observed that in a cohort of older patients affected by metastatic castration-resistant prostate cancer (mCRPC) treated with ARPIs, the percentage of non-adherence to ARPIs was relatively low (4.5%) and it was related to a frailty condition identified by a low score at the geriatric G8 screening questionnaire^7^. The frailty condition in the older population was investigated through a comprehensive geriatric assessment tool including tests and scales such as the geriatric G8, IADL, and the Charlson comorbidity scales alongside an assessment of the presence and characteristics of the caregiver. The reliability of these scales has already been validated in older cancer patients, including those suffering from urological cancers^8–11^. Taking advantage of the above-mentioned results, the role of the IADL scale was emphasized.

IADL is a significant health indicator with items closely related to executive functions since it includes items able to detect mild cognitive impairment such as taking medication^12,13^*.* Whereas the ADL scale primarily relies on physical and health-related factors, the IADL scale is strongly related to cognitive resources. Moreover, a decline in IADL usually precedes limitations in ADL, and may therefore predict a cognitive decline^14^.

Despite caregivers frequently providing significant amounts of care and support to cancer patients, few studies have explored the relationship between caregiver contribution to adherence to oral anticancer treatment and outcomes^15^.

The Meet-URO 5—ADHERE study was a prospective multicentre observational cohort study monitoring adherence to androgen receptor pathway inhibitors (ARPI) in 234 metastatic castrate-resistant PC (mCRPC) patients aged ≥ 70. We found an association between the geriatric G8 assessment and adherence to ARPI [Rescigno 2022], but also with radiographic progression-free survival (rPFS) and overall survival (OS)^16^.

In the current analysis, we aimed to investigate the association of patients’ IADL scores with other clinical factors, with a particular focus on the presence of a caregiver, and the impact on adherence to ARPIs and survival outcomes.

## Methods

The Meet-URO 5 ADHERE study was a prospective multicentre observational trial conducted in 6 centres joining the Meet-Uro Italian Network for Research in Urologic Oncology, in which older mCRPC patients (≥ 70 years) were monitored to assess adherence to abiraterone or enzalutamide. The study protocol is described in the original publication^7^ and was centrally approved by the Catania-1 ethical committee (n.12/2019/CA, 15 February 2019). All 234 enrolled patients between February 2019 and September 2021 signed the protocol informed consent.

At the screening visit, patients underwent the geriatric G8, Charlson comorbidity and IADL assessments and a 5-item caregiver evaluation questionnaire was administered to the patient. The patient was asked about the presence, identification, and characteristics of the caregiver.

Adherence to ARPIs was assessed by pill counting and patient self-reporting through a modified and adapted basel assessment of adherence scale (BAAS)^7,17,18^. For non-adherence to ARPIs, the assessment was by pill-counting, a cut-off of 5% was used based on the median non-adherence detected in the original study^7^.

The current analysis hypothesised that an impaired IADL was associated with the presence of a caregiver and had a negative impact on adherence to ARPIs and survival outcomes. The presence of the caregiver in older mCRPC patients receiving ARPIs has already been investigated and reported by our research group in a previous analysis of the same study^11^, highlighting its association with patients’ older age, lower geriatric G8 score, a detrimental effect in rPFS, and a trend toward worse OS.

Three categories of IADL score were used for the analysis: < 6, 6–7, and ≥ 8. For the other study clinical variables, the literature reported, or the cut-offs identified in our previous analyses were used^16^. Particularly, the unsupervised median value threshold of 10 for the Charlson comorbidity score, 31 months for the time to castration-resistant disease, and 80 years for older age, were preferred to the literature-reported values of 9^19^, < 12 months^20^, and ≥ 75, respectively. This was based on their lowest p-values shown at the previous UVA^16^.

Differences in socio-demographical and clinical characteristics across IADL levels were evaluated through Fisher’s exact test. The risk of non-adherence, expressed as odds ratio (OR) and corresponding 95% confidence interval (CI), was estimated through a univariable unconditional logistic regression model (UVA) for the following clinical characteristics: age (< 80 vs ≥ 80 years), Gleason score (< 8 vs ≥ 8), time to castration resistance (≥ 31 vs < 31 months [mo.]), sites of metastases (lymph nodes only vs bone vs other), setting of therapy (pre- vs post-chemotherapy vs post-abiraterone or enzalutamide), Charlson comorbidity score (< 10 vs ≥ 10), geriatric G8 (≥ 14 vs < 14), caregiver presence (absent vs present); ARPI type (abiraterone vs enzalutamide), number of previous chemotherapy lines (0 vs 1 vs ≥ 2), PSA decline by 50% (PSA50) (no vs yes), and IADL (< 6, 6–7, and ≥ 8). All clinical characteristics with p < 0.05 at UVA were included in the multivariable analysis (MVA), i.e. Gleason score, caregiver presence, and IADL level.

For each patient, the time at risk for rPFS was calculated from ARPI start to date of disease progression on imaging as per RECIST 1.1, death from any cause, or last follow-up, whichever occurred first. Time at risk for OS was calculated from the ARPI start date until death or the last follow-up. Survival probabilities for rPFS and OS were estimated through the Kaplan–Meier method and differences between curves were tested using the log-rank test.

Cox regression UVA and MVA analyses were conducted to evaluate the hazard ratio (HR) of death or progression, by IADL level alone and in combination with caregiver presence. MVA included all clinical characteristics significantly associated with OS/PFS at UVA.

The analysis was performed using the statistical software SAS 9.4.

### Ethics approval

The study was approved by the Catania-1 ethical committee (n.12/2019/CA of the 15th of February 2019). The study was performed in accordance with the Good Clinical Practice guidelines of the International Conference on Harmonization and the Declaration of Helsinki.

### Consent to participate

All patients provided the protocol written informed consent.

## Results

### Patients’ socio-demographic characteristics associated with IADL

The main characteristics of patients according to the three IADL score categories (< 6, 6–7, ≥ 8) are summarised in Table 1. There was a significant association between the geriatric G8 score and the three categories of IADL (p < 0.01); patients with a better geriatric G8 score (i.e. ≥ 14) were less represented in the < 6 and 6–7 IADL categories. No other significant association between the IADL score categories and other socio-demographic patient characteristics were found including older age (with a cut-off of 80 years, p = 0.18), metastatic site (p = 0.75), Charlson comorbidity score (p = 0.15) and caregiver presence (p = 0.46).

**Table 1:** Clinical characteristics by IADL.

	All patients	IADL			Fisher’s exact test
		< 6	6–7	≥ 8	
	n (%)	n (%)	n (%)	n (%)	
Age (years)					
< 80	150 (64.1)	43 (55.8)	53 (67.1)	54 (69.2)	p = 0.18
≥ 80	84 (35.9)	34 (44.2)	26 (32.9)	24 (30.8)	
Gleason score					
< 8	78 (36.4)	26 (38.8)	20 (27.0)	32 (43.8)	p = 0.09
≥ 8	136 (63.6)	41 (61.2)	54 (73.0)	41 (56.2)	
Missing	20				
Time to CR (months)					
≥ 31	118 (50.4)	37 (48.1)	41 (51.9)	40 (51.3)	p = 0.89
< 31	116 (49.6)	40 (51.9)	38 (48.1)	38 (48.7)	
Site of mets					
Lymph nodes (only)	49 (20.9)	13 (16.9)	17 (21.5)	19 (24.3)	p = 0.75
Bone (non-visceral)	163 (69.7)	57 (74.0)	53 (67.1)	53 (68.0)	
Other	22 (9.4)	7 (9.1)	9 (11.4)	6 (7.7)	
Setting					
Post-ChT	57 (24.4)	14 (18.2)	19 (24.1)	24 (30.8)	p = 0.39
Pre-ChT	162 (69.2)	58 (75.3)	56 (70.9)	48 (61.5)	
Post-Abi/Enza	15 (6.4)	5 (6.5)	4 (5.1)	6 (7.7)	
Charlson comorbidity score					
< 10	59 (25.2)	14 (18.2)	25 (31.7)	20 (25.6)	p = 0.15
≥ 10	175 (74.8)	63 (81.8)	54 (68.4)	58 (74.4)	
Geriatric G8					
≥ 14	87 (37.2)	16 (20.8)	28 (35.4)	43 (55.1)	p < 0.01
< 14	147 (62.8)	61 (79.2)	51 (64.6)	35 (44.9)	
Caregiver					
Absent	44 (18.8)	14 (18.2)	12 (15.2)	18 (23.1)	p = 0.46
Present	190 (81.2)	63 (81.8)	67 (84.8)	60 (76.9)	
Treatment					
Abi	86 (36.8)	25 (32.5)	31 (39.2)	30 (38.5)	p = 0.64
Enza	148 (63.2)	52 (67.5)	48 (60.8)	48 (61.5)	
Number of previous ChT lines					
0	156 (66.7)	53 (68.8)	53 (67.1)	50 (64.1)	p = 0.79
1	60 (25.6)	19 (24.7)	18 (22.8)	23 (29.5)	
≥ 2	18 (7.7)	5 (6.5)	8 (10.1)	5 (6.4)	
PSA50					
No	65 (27.8)	18 (23.7)	23 (29.9)	24 (31.6)	p = 0.51
Yes	164 (72.2)	58 (76.3)	54 (70.1)	52 (68.4)	

*Abi* abiraterone, *CR* castration-resistance, *ChT* chemotherapy, *Enza* enzalutamide, *IADL* instrumental activities of daily living; mets, metastases, *PSA50* PSA decline ≥ 50% from the baseline value.

### Adherence to ARPIs according to IADL

Adherence to ARPIs, either assessed by pill counting or patient reporting, was significantly associated with the three IADL categories (p = 0.01 and p = 0.03, respectively; Table 2).

**Table 2:** Treatment adherence according to IADL.

	All patients	IADL			Fisher’s exact test
		< 6	6–7	≥ 8	
	n (%)	n (%)	n (%)	n (%)	
Pills count adherence					
> 95%	153 (65.4)	40 (51.9)	57 (72.1)	56 (71.8)	p = 0.01
≤ 95%	81 (34.6)	37 (48.1)	22 (27.9)	22 (28.2)	
Adapet BAAS					
Adherent	175 (74.8)	60 (77.9)	51 (64.6)	64 (82.0)	p = 0.03
Not adherent	59 (25.2)	17 (22.1)	28 (35.4)	14 (18.0)	

*BAAS* basel assessment of adherence scale, *IADL* instrumental activities of daily living.

By pill counting, 34.6% of patients adhered to ≤ 95% of the ARPI prescribed dose. Those in the < 6 IADL category were less adherent (48.1%) than the ones with 6–7 (27.9%) and ≥ 8 (28.2%) IADL scores.

By patient-reported adherence, 25.2% of patients reported non-adherence to the prescribed dose of ARPI. A higher proportion of patients in the 6–7 and < 6 IADL categories (35.4% and 22.1%) reported being non-adherent than ≥ 8 IADL (18%).

### Factors affecting adherence to ARPIs and IADL

At the UVA, factors significantly associated with ARPIs adherence were Gleason score ≥ 8 (OR = 0.40, 95% CI 0.22–0.72, p < 0.01), the presence of a caregiver (OR = 0.36, 95% CI 0.18–0.70, p < 0.01) and IADL < 6 (OR = 2.35, 95% CI 1.21–4.58, p = 0.01 – Supplementary Table 1). When a combination of IADL with a threshold of 6 and caregiver presence was analysed, the worst risk factor for reduced adherence to ARPIs was IADL < 6 with the absence of a caregiver (OR = 11.36, 95% CI 2.98–43.3, p < 0.01). Older age (≥ 80 years), time to castration resistance, metastatic sites, ARPI setting and type, number of previous chemotherapy lines and PSA response to ARPI did not result as adverse factors for adherence to ARPIs (see Supplementary Table 1).

**Figure 1:**
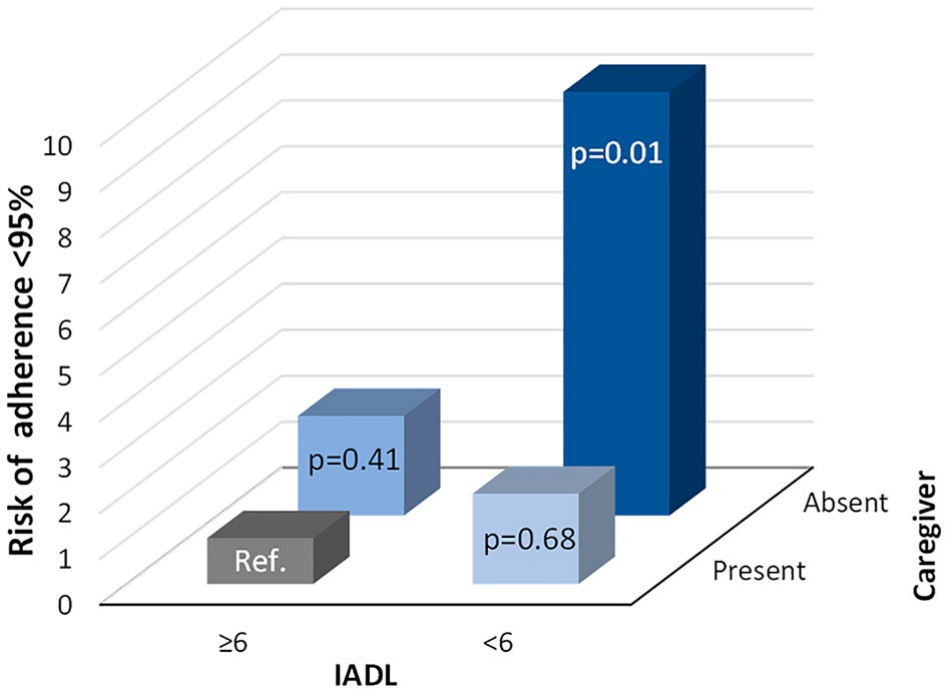
Interaction between IADL with a threshold of 6 and caregiver presence. *IADL* instrumental activities of daily living.

A Gleason score ≥ 8 (OR = 0.37, 95% CI 0.20–0.69, p < 0.01), the presence of caregiver (OR = 0.36, 95% CI 0.18–0.76, p < 0.01), and IADL < 6 (OR 2.55, 95% CI 1.22–5.36, p = 0.01) were confirmed as independent factors for adherence to ARPIs at the MVA, alongside the combination of IADL < 6 and the absence of a caregiver (OR 9.23, 95% CI 2.28–37.43, p = 0.01) (see Supplementary Table 1 and Fig. 1).

### IADL and survival outcomes

**Figure 2:**
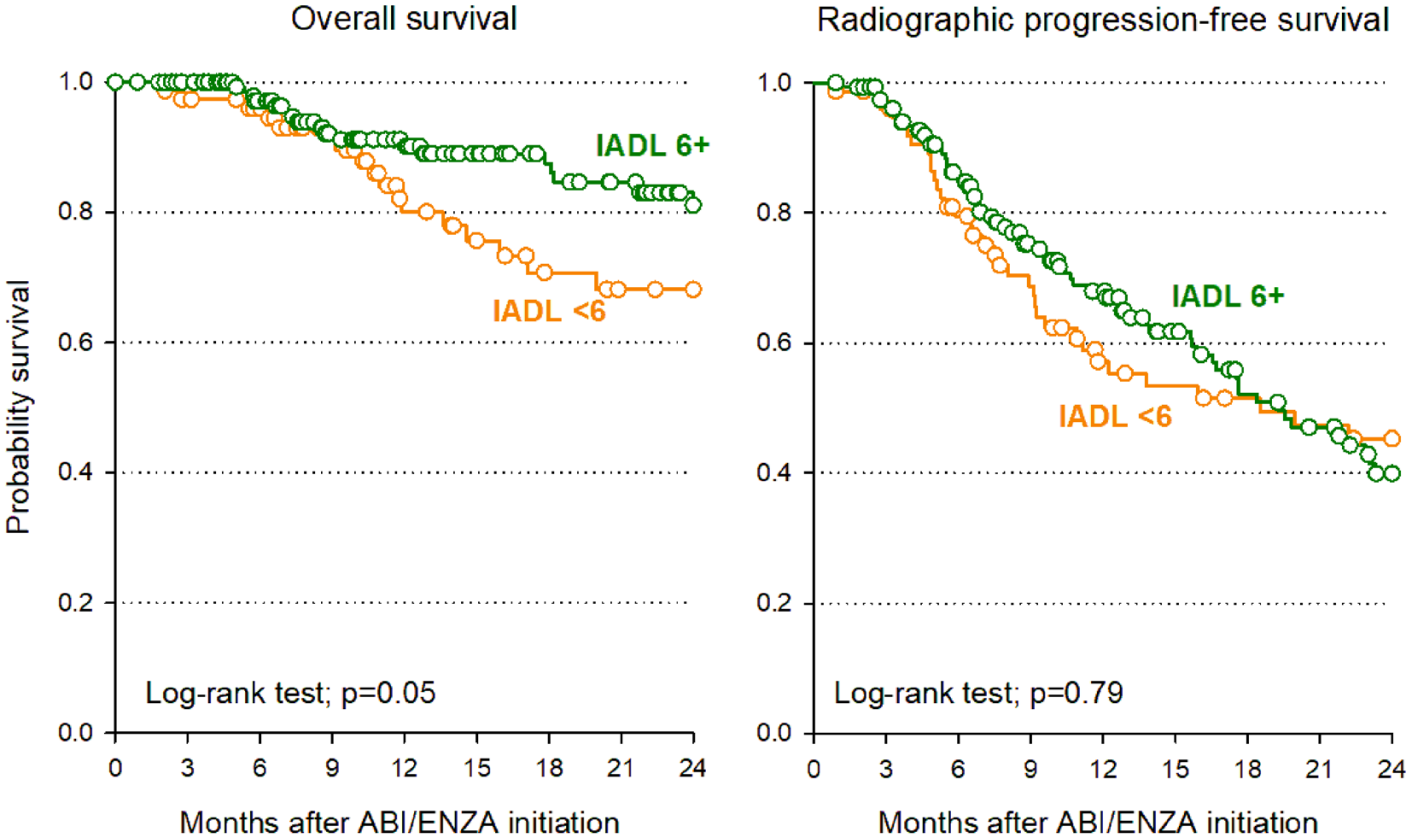
Kaplan–Meier overall survival and radiographic progression-free survival estimates according to IADL with a threshold of 6. *ABI* abiraterone, *ENZA* enzalutamide, *IADL* instrumental activities of daily living.

Patients with IADL < 6 had a trend toward worse OS (p = 0.05) but no different rPFS (p = 0.79) compared to IADL ≥ 6 (see Fig. 2).

Combining the IADL with a threshold of 6 with caregiver presence, a non-significant toward worse OS was observed in patients with IADL < 6 and caregiver (p = 0.10), whereas non-significantly better rPFS was found in those with IADL ≥ 6 and no caregiver (p = 0.05) (see Supplementary Fig. 1), although this combination resulted at MVA as one of the independent prognostic factors for rPFS (HR 0.27, 95% CI 0.10–0.74, p = 0.01) (see Supplementary Table 2).

## Discussion

The International Society of geriatric oncology (SIOG) task force recommends taking treatment decisions in older prostate cancer patients besides their chronological age and suggests performing functional status assessment beyond the Eastern cooperative oncology group performance status (PS) evaluation^21^. This includes geriatric G8 and IADL scores as surrogates for a more time-consuming comprehensive geriatric assessment (CGA). Dependence is another key factor contributing towards a poor PS; therefore, it should also be enquired whether patients need or have a caregiver as baseline assessment before starting an anticancer treatment^21^.

IADL involves activities with high levels of functional ability, cognition, and judgment, necessary to maintain independent living, well-being, and achieving an optimal balance between health and quality of life (QoL). They include household chores, self-care, meal preparation, shopping, financial management, and the use of public transportation^22^. Cancer patients tend to have reduced IADL, with nearly half of them needing help to perform daily activities, as assessed in a comprehensive meta-analysis of 19.246 patients^23^. However, different types of cancers affecting primarily older patients might have a different impact on IADL. Breast cancer has been associated with reduced ability to work^24^, while older patients with haematological cancers might have reduced performance in IADL, requiring help in daily activities or hospitalization^25^.

The IADL scale is an easy tool to administer (it takes about 10–15 min to complete), and it consists of eight items. Each ability investigated relies on cognitive and/or physical function, though all require some degree of both^26^. IADL is associated with cognitive aspects and functions. Indeed, a decline in functions and impairment in daily activities are signs of early dementia^27,28^. Moreover, IADL function is usually impaired before the loss of ADL function (such as eating, bathing, and using the toilet). Therefore, assessing IADLs might be able to identify incipient physical and/or cognitive decline in an older adult who otherwise appears healthy^29^. As such, a comprehensive assessment of IADL and geriatric or cognitive aspects might be of paramount importance to evaluate older cancer patients to predict their adherence to cancer treatments and clinical outcomes.

Randomized and controlled trials (RCTs) on mCRPC often include QoL and the impact of interventions or cancer on IADL as secondary outcomes^30^. Nevertheless, older adults are under-represented in clinical trials rendering it arduous to gather such data in this population. Moreover, older adults represent a heterogenous population, including both fit and vulnerable individuals, who are however treated as younger men in standard practice^31^, and even fewer prospective data exist on IADL/QOL on older mCRPC patients treated as per standard of care.

We have previously shown that the geriatric G8 score is associated with adherence to ARPIs in patients with mCRPC and with clinical outcomes^7,16^. Here, we observed an association between IADL and adherence to ARPIs, pointing to a reduced capacity to take prescribed medications in older men with impaired IADL. Moreover, we observed that older patients with an IADL score < 6 and without a caregiver had the highest risk of nonadherence to ARPI. This group represents a highly vulnerable population. In the original study analysis^7^, we were unable to establish a connection between non-adherence to ARPIs and survival outcomes in our older population of mCRPC patients. This finding may, in fact, support the seemingly contradictory observation of higher non-adherence but better survival outcomes in the subgroup of patients with IADL < 6 and no caregiver, compared to those with IADL < 6 and supported by a caregiver. Additionally, as we have previously reported within the same study^32^, the presence of a caregiver was found to be associated with patients’ older age, lower geriatric G8 score, a detrimental effect on rPFS, as well as a potential trend towards worse OS. It is therefore plausible that the presence of caregivers in our study reflected worse patients’ cognitive and social functioning within the same IADL score < 6 score group. This suggests the presence of more severe impairments requiring caregiver assistance, further distinguishing patients with IADL scores < 6 score and potentially impacting survival outcomes negatively.

An association between IADL and geriatric G8 scores was also documented. Rier and colleagues studied 291 patients aged ≥ 65 years treated with chemotherapy for any cancer type in a prospective cohort study. CGA and the geriatric G8 screening assessments were performed before and after the completion of chemotherapy. They found that an abnormal geriatric G8 score before chemotherapy was a risk factor for a decline of IADL-independence (OR 3.60, 95% CI 1.98–6.54, p < 0.0001), prematurely terminated chemotherapy (OR 2.12, 95% CI 1.24–3.65, p = 0.006), and shorter median OS (HR 1.71, 95% CI 1.16–2.52, p = 0.007) especially in patients with solid malignancies^33^. These data, as well as our analysis, confirm the interdependence between IADL and geriatric G8 score, and how much the impairment in daily activity and cognition might impact treatment and clinical outcomes in older cancer patients.

We did not find a significant association between impaired IADL alone or in combination with the presence of a caregiver and survival outcomes (i.e. rPFS and OS). However, although pre-planned, the analysis was not powered to study survival differences in the presented subgroups. Prospective ad-hoc studies are warranted to better study the role of IADL and geriatric G8 score assessments in older mCRPC patients.

The reason behind the higher adherence to ARPIs in patients with a Gleason score ≥ 8 is challenging to comprehend; even though we cannot rule out the possibility that the patients’ perceived severity of the disease might lead to a stronger commitment to ARPIs, further research is needed to uncover the underlying factors influencing this trend.

In conclusion, study findings suggest that IADL score and geriatric G8 scales represent optimal tools to identify less auto-sufficient patients who have the highest risk of nonadherence to ARPIs. if not supported by a caregiver and sheds light on a more vulnerable group that might need more follow-up.

### Supplementary Information

Supplementary Information.

## Data Availability

The datasets used and/or analysed during the current study available from the corresponding author on reasonable request.
