# Supplementary material for: Instrumental activities of daily living in older patients with metastatic prostate cancer: results from the meet-URO network ADHERE prospective study
Source: Sci Rep. 2024 Feb 28;14:4949. doi: 10.1038/s41598-024-53581-4 (PMC10902368; doi:10.1038/s41598-024-53581-4)
Supplement: Supplementary file 1 — Supplementary Information. [file 41598_2024_53581_MOESM1_ESM.docx]

**Supplementary figure 1**. Kaplan-Meier overall survival and radiographic progression-free survival estimates according to IADL with threshold of 6 and caregiver presence.

Abbreviations: Abi, abiraterone; Enza, enzalutamide

**Supplementary Table 1**. Univariable and multivariable analysis of factors affecting treatment adherence assessed by pill-counting

|  | **Adherence** | | | | **Univariate**  **OR (95% CI)** | **p** | **Multivariable^a^**  **OR (95% CI)** | **p** |
| --- | --- | --- | --- | --- | --- | --- | --- | --- |
|  | **≤95%** | | **>95%** | |  |  |  |  |
|  | **n** | **(%)** | **n** | **(%)** |  |  |  |  |
| Age (years) |  |  |  |  |  |  |  |  |
| <80 | 47 | (31.3) | 103 | (68.7) | Reference |  |  |  |
| ≥80 | 34 | (40.5) | 50 | (59.5) | 1.49 (0.86-2.60) | 0.16 |  |  |
| Gleason score |  |  |  |  |  |  |  |  |
| <8 | 37 | (47.4) | 41 | (52.6) | Reference |  | Reference |  |
| ≥8 | 36 | (26.5) | 100 | (73.5) | 0.40 (0.22-0.72) | <0.01 | 0.37 (0.20-0.69) | <0.01 |
| Time to CR (months) | | |  |  |  |  |  |  |
| ≥31 | 44 | (37.3) | 74 | (62.7) | Reference |  |  |  |
| <31 | 37 | (31.9) | 79 | (68.1) | 0.79 (0.46-1.35) | 0.39 |  |  |
| Site of mets | | | | | |  |  |  |
| Lymph nodes | 16 | (32.7) | 33 | (67.4) | Reference |  |  |  |
| Bone | 55 | (33.7) | 108 | (66.3) | 1.05 (0.53-2.07) | 0.48 |  |  |
| Other | 10 | (45.5) | 12 | (54.6) | 1.72 (0.61-4.81) | 0.26 |  |  |
| Setting |  |  |  |  |  |  |  |  |
| Post-ChT | 16 | (28.1) | 41 | (71.9) | Reference |  |  |  |
| Pre-ChT | 36 | (36.4) | 103 | (63.6) | 1.46 (0.76-2.84) | 0.25 |  |  |
| Post-Abi/Enza | 6 | (40.0) | 9 | (60.0) | 1.71 (0.52-5.58) | 0.38 |  |  |
| Charlson comorbidity score | | |  |  |  |  |  |  |
| <10 | 18 | (30.5) | 41 | (69.5) | Reference |  |  |  |
| ≥10 | 63 | (36.0) | 112 | (64.0) | 1.28 (0.68-2.42) | 0.44 |  |  |
| Geriatric G8 |  |  |  |  |  |  |  |  |
| ≥14 | 25 | (28.7) | 62 | (71.3) | Reference |  |  |  |
| <14 | 56 | (38.1) | 91 | (61.9) | 1.53 (0.86-2.70) | 0.15 |  |  |
| Treatment |  |  |  |  |  |  |  |  |
| Abi | 31 | (36.1) | 55 | (64.0) | Reference |  |  |  |
| Enza | 50 | (33.8) | 98 | (66.2) | 0.91 (0.52-1.58) | 0.73 |  |  |
| Number of previous ChT lines | | |  |  |  |  |  |  |
| 0 | 58 | (37.2) | 98 | (62.8) | Reference |  |  |  |
| ≥1 | 23 | (29.5) | 55 | (70.5) | 0.71 (0.39-1.27) | 0.24 |  |  |
| PSA50 |  |  |  |  |  |  |  |  |
| No | 24 | (36.9) | 41 | (63.1) | Reference |  |  |  |
| Yes | 55 | (33.5) | 109 | (66.5) | 0.86 (0.47-1.57) | 0.63 |  |  |
| Caregiver |  |  |  |  |  |  |  |  |
| Absent | 24 | (54.6) | 20 | (45.6) | Reference |  | Reference |  |
| Present | 57 | (30.0) | 133 | (70.0) | 0.36 (0.18-0.70) | <0.01 | 0.36 (0.18-0.76) | <0.01 |
| IADL |  |  |  |  |  |  |  |  |
| ≥8 | 22 | (28.2) | 56 | (71.8) | Reference |  | Reference |  |
| 6-7 | 22 | (27.9) | 57 | (72.1) | 0.98 (0.49-1.97) | 0.96 | 1.27 (0.59-2.70) | 0.48 |
| <6 | 37 | (48.0) | 40 | (52.0) | 2.35 (1.21-4.58) | 0.01 | 2.55 (1.22-5.36) | 0.01 |
| Caregiver and IADL | | |  |  |  |  |  |  |
| Present, IADL≥6 | 31 | (24.4) | 96 | (75.6) | Reference |  |  |  |
| Present, IADL<6 | 26 | (41.3) | 37 | (58.7) | 2.18 (1.14-4.15) | 0.02 | 1.97 (0.98-3.96) | 0.41 |
| Absent, IADL≥6 | 13 | (43.3) | 17 | (56.7) | 2.37 (1.04-5.42) | 0.04 | 2.17 (0.91-5.18) | 0.68 |
| Absent, IADL<6 | 11 | (78.6) | 3 | (21.4) | 11.36 (2.98-43.33) | <0.01 | 9.23 (2.28-37.43) | 0.01 |

Abbreviations: Abi, abiraterone; CI, confidence interval; CR, castration-resistance; ChT, chemotherapy; Enza, enzalutamide; IADL, instrumental activities of daily living; mets, metastases; OR, odds ratio; PSA50, PSA decline ≥50% from the baseline value.

^a^Including all significant variables at the univariate analysis, excluding caregiver and IADL.

**Supplementary Table 2**. Univariable and multivariable analysis of factors affecting overall survival and radiographic progression-free survival

|  | **Patients** | **Overall survival** | | | | | |  | **Radiographic Progression-free survival** | | | | | |
| --- | --- | --- | --- | --- | --- | --- | --- | --- | --- | --- | --- | --- | --- | --- |
|  |  | **Events** | | **Univariate** | | **Multivariable^a^** | |  | **Events** | | **Univariate** | | **Multivariable^a^** | |
|  |  | **n** | **(%)** | **HR (95% CI)** | **p** | **HR (95% CI)** | **p** |  | **n** | **(%)** | **HR (95% CI)** | **p** | **HR (95% CI)** | **p** |
| Age (years) |  |  |  |  |  |  |  |  |  |  |  |  |  |  |
| <80 | 150 | 16 | (10.7) | Reference |  | Reference |  |  | 65 | (43.3) | Reference |  |  |  |
| ≥80 | 84 | 19 | (22.6) | 2.52 (1.29-4.90) | <0.01 | 2.41 (1.16-4.96) | 0.02 |  | 37 | (44.1) | 1.14 (0.76-1.72) | 0.53 |  |  |
| Number of previous ChT lines | | | | | | | | | | | | | | |
| 0-1 | 216 | 32 | (14.8) | Reference |  |  |  |  | 89 | (41.2) | Reference |  | Reference |  |
| ≥2 | 18 | 3 | (16.7) | 1.78 (0.54-5.82) | 0.34 |  |  |  | 13 | (72.2) | 2.53 (1.38-4.64) | <0.01 | 2.26 (1.14-4.45) | 0.02 |
| Site of mets |  |  |  |  |  |  |  |  |  |  |  |  |  |  |
| Lymph nodes | 49 | 0 | (0.0) | - |  |  |  |  | 7 | (14.3) | Reference |  | Reference |  |
| Other | 185 | 35 | (18.9) | - |  |  |  |  | 95 | (51.4) | 4.12 (1.91-8.89) | <0.01 | 3.88 (1.66-9.06) | <0.01 |
| Gleason score |  |  |  |  |  |  |  |  |  |  |  |  |  |  |
| <8 | 78 | 9 | (11.5) |  |  |  |  |  | 25 | (32.1) | Reference |  | Reference |  |
| ≥8 | 136 | 23 | (16.9) | 1.67 (0.77-3.61) | 0.19 |  |  |  | 67 | (49.3) | 1.93 (1.22-3.06) | <0.01 | 1.86 (1.14-3.03) | 0.01 |
| Time to CR (months) | | | | | | | | | | | | | | |
| ≥31 | 118 | 13 | (11.0) | Reference |  | Reference |  |  | 41 | (34.8) | Reference |  | Reference |  |
| <31 | 116 | 22 | (19.0) | 2.27 (1.14-4.52) | 0.02 | 3.22 (1.59-6.54) | <0.01 |  | 61 | (52.6) | 2.03 (1.36-3.03) | <0.01 | 2.24 (1.45-3.47) | <0.01 |
| Treatment |  |  |  |  |  |  |  |  |  |  |  |  |  |  |
| Abi | 86 | 12 | (14.0) | Reference |  |  |  |  | 34 | (39.5) | Reference |  |  |  |
| Enza | 148 | 23 | (15.5) | 1.26 (0.63-2.53) | 0.52 |  |  |  | 68 | (46.0) | 1.38 (0.91-2.09) | 0.13 |  |  |
| Surgery |  |  |  |  |  |  |  |  |  |  |  |  |  |  |
| No | 146 | 25 | (17.1) | Reference |  |  |  |  | 69 | (47.3) | Reference |  | Reference |  |
| Yes | 88 | 10 | (11.4) | 0.49 (0.23-1.02) | 0.06 |  |  |  | 33 | (37.5) | 0.61 (0.40-0.93) | 0.02 | 0.98 (0.62-1.57) | 0.94 |
| Geriatric G8 |  |  |  |  |  |  |  |  |  |  |  |  |  |  |
| ≥14 | 87 | 6 | (6.9) | Reference |  | Reference |  |  | 30 | (34.5) | Reference |  | Reference |  |
| <14 | 147 | 29 | (19.7) | 3.30 (1.37-7.96) | <0.01 | 2.82 (1.11-7.19) | 0.03 |  | 72 | (49.0) | 1.68 (1.08-2.59) | 0.02 | 1.67 (1.05-2.66) | 0.03 |
| Caregiver |  |  |  |  |  |  |  |  |  |  |  |  |  |  |
| Absent | 44 | 3 | (6.8) | Reference |  |  |  |  | 10 | (22.7) | Reference |  | Reference |  |
| Present | 190 | 32 | (16.8) | 2.52 (0.77-8.25) | 0.13 |  |  |  | 92 | (48.4) | 2.29 (1.19-4.40) | 0.01 | 2.10 (1.05-4.19 | 0.04 |
| IADL |  |  |  |  |  |  |  |  |  |  |  |  |  |  |
| ≥6 | 157 | 18 | (7.7) | Reference |  |  |  |  | 25 | (32.1) | Reference |  |  |  |
| <6 | 77 | 17 | (22.1) | 1.90 (0.98-3.67) | 0.06 |  |  |  | 36 | (46.8) | 1.06 (0.70-1.60) | 0.79 |  |  |
| Caregiver / IADL |  |  |  |  |  |  |  |  |  |  |  |  |  |  |
| Present / ≥6 | 127 | 17 | (13.4) | Reference |  |  |  |  | 61 | (48.2) | Reference |  | Reference |  |
| Present / <6 | 63 | 15 | (23.8) | 1.76 (0.88-3.52) | 0.11 |  |  |  | 31 | (49.2) | 0.93 (0.60-1.44) | 0.74 | 0.85 (0.52-1.39) | 0.53 |
| Absent / ≥6 | 30 | 1 | (3.3) | 0.24 (0.03-1.84) | 0.17 |  |  |  | 5 | (16.7) | 0.30 (0.12-0.75) | <0.01 | 0.27 (0.10-0.74) | 0.01 |
| Absent / <6 | 14 | 2 | (14.3) | 1.03 (0.24-4.44) | 0.97 |  |  |  | 5 | (35.7) | 0.74 (0.30-1.85) | 0.52 | 1.04 (0.41-2.65) | 0.93 |

Abbreviations: Abi, abiraterone; CI, confidence interval; CR, castration-resistance; ChT, chemotherapy; Enza, enzalutamide; HR, hazard ratio; IADL, instrumental activities of daily living; mets, metastases.

^a^Including all significant variables at the univariate analysis.
